# Postoperative improvement in the index finger center of pressure trajectory during precision grip in patients with degenerative cervical myelopathy

**DOI:** 10.1371/journal.pone.0328197

**Published:** 2025-08-01

**Authors:** Duy Quoc Vo, Lu Zhang, Naoto Noguchi, Ken Kondo, Ryoto Akiyama, Hiroshi Fujii, Yuki Obokata, Bumsuk Lee

**Affiliations:** 1 Gunma University Graduate School of Health Sciences Doctoral Program, Maebashi, Gunma, Japan; 2 Gunma University Graduate School of Health Sciences, Maebashi, Gunma, Japan; 3 Department of Occupational Therapy Faculty of Rehabilitation, Gunma Paz University, Takasaki, Gunma, Japan; 4 Rehabilitation Center, East Maebashi Orthopedic Hospital, Maebashi, Gunma, Japan; Tokyo Medical and Dental University (TMDU), JAPAN

## Abstract

**Objective:**

The aim of this study was to kinetically evaluate the changes in precision grip in patients with degenerative cervical myelopathy before and after decompressive surgery.

**Methods:**

Twenty-nine patients with degenerative cervical myelopathy participated in the study. Participants performed a grip-and-lift task by pulp pinch using their thumb and index finger before and after surgery. We monitored individual finger grip force (GF) and center of pressure (COP) trajectory in total five seconds and during the first second while lifting and holding an object. Correlations between the pre-operative COP trajectory and other hand clinical outcomes were analyzed. A multiple regression analysis was used to identify the predictive value of the pre-operative COP trajectory to the severity of sensorimotor dysfunction.

**Results:**

There was a significant improvement in the 1-second COP trajectory in the index finger after surgery, but not the GF. Moreover, pre-operative COP trajectory was associated with the post-operative severity of clinical symptoms. The multiple regression analysis concerning the severity of upper extremity symptoms indicated that the model incorporating pre-operative COP trajectory exhibited the highest adjusted R^2^ compared to GF or conventional clinical tests.

**Conclusion:**

These results suggested that patients with degenerative cervical myelopathy could improve their COP trajectory in the index finger after surgery, and the finger kinetic measure could provide an important index for predicting neurological improvement.

## Introduction

Degenerative cervical myelopathy (DCM), a neurological condition, mostly happens in the elderly due to compression of the spinal cord in the neck region. It can result in a range of symptoms, including neck pain, numbness or tingling in the extremities, loss of coordination, and even paralysis [1]. Cervical decompressive surgery is recommended in severe cases where conservative treatments fail, or when neurological deficits are present.

One of the top ten research priorities for DCM is to monitor post-surgical functional improvement in neurological impairments [2]. In line with the priority, assessing upper limb function has become increasingly important for rehabilitation, particularly for activities of daily living independence. Numerous assessments on hand functions have been proposed and applied to follow the improvement of patients with DCM after surgery investigating hand functions such as finger/hand strength, finger coordination, or hand dexterity [3–7]. For example, the 10-second grip-and-release test (10-s G&R) and the nine-hole peg test (9HPT) are commonly applied and have shown effectiveness in detecting and monitoring DCM [8]. However, the assessments focus only on how participants complete the tasks, leaving the instability of finger motions inexplicable. While the 10-s G&R test and 9HPT are simple to administer, they can be prone to interobserver variability when assessed visually. To address this, it requires upgraded, kinetic parameters to deal with the limitations of the original tests.

Grip force (GF) and center of pressure (COP) are the important kinetic parameters of precision grip performance. The research on GF control is expected to explain what causes finger dysfunction at the basic level [9]. A comprehensive understanding of GF control might contribute to the appropriate selection of rehabilitative programs and hand therapy approaches [10]. On the other hand, the finger COP is used as the kinetic factor in GF control, representing how forces produced by the thumb and index finger in precision grip are balanced on the contact surfaces [9,11,12].

Although the importance of the two kinetic parameters in precision grip is recognized, there is limited information about postoperative changes in patients with DCM. To our knowledge, it is only known that GF control is associated with daily hand activities in preoperative patients with DCM [10,13]. In COP trajectory, no existing study is investigating this population either in the preoperative or postoperative period. To date, GF control and COP trajectory have not been comprehensively investigated in patients with DCM before and after surgery.

We aimed to extend our understanding of the characteristics of GF control and COP trajectory in patients with DCM before and after surgery. We hypothesized that (1) the patients might have a change of their GF control and COP trajectory in the grip-and-lift task, and (2) preoperative GF and COP trajectory would be the sensible indices to early predict the outcomes of the surgery.

## Methods

### Subjects

Twenty-nine patients diagnosed with DCM (20 males and 9 females, 70.2 ± 6.8 years) at a local hospital were recruited in the study. This prospective study was conducted from April 25, 2022 to November 29, 2023. The patients participated at the day before receiving decompressive surgery and the day of discharge with the follow-up time averaging 29.4 ± 21.4 days. The median duration of symptoms was 21.7 ± 21.1months, and all participants received laminoplasty at C3-7 levels. The result from Japanese-version Edinburgh Handedness Inventory indicated all patients were right-handed. They were able to walk with or without walking aid and were normal or correct-to-normal vision. The inclusion criteria were (1) confirmed to have DCM by magnetic resonance imaging (MRI) and clinical symptoms and signs of DCM without any history of cervical spine surgery; (2) ability to perform the holding task with the right thumb and index finger; and (3) normal cognitive functions to understand the commands and follow the requirements of any clinical tests. The exclusion criteria were (1) comorbid with cervical radiculopathy, and (2) any physical and psychological disorders that might impair the grip-and-lift task performance. Written informed consent was obtained from each individual prior to the experiment. This study involving human participants was performed in line with the principles of the 1964 Helsinki Declaration, and the study was approved by the Ethical Review Board of the East Maebashi Orthopedic Hospital, and Gunma University Ethical Review Board for Medical Research Involving Human Subject (HS2021−028).

### Grip-and-lift task

**Fig 1:**
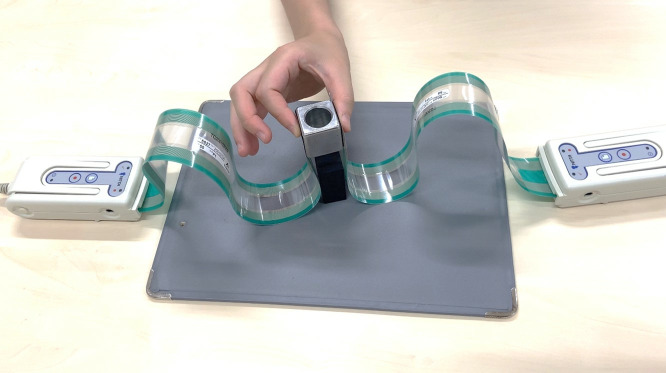
The grip-and-lift task. Participants used the thumb and index finger pulps of the dominant hand to grip and lift a 150 g iron cube with the two pressure sensor sheets attached to the grip surface sides.

The experimental procedure was in accordance with the previous study by Johansson and Westling [14]. The participants were seated on a chair facing towards a height-adjustable table. Prior to the task, the finger pads were cleansed with alcohol swabs to reduce oil and sweat which might affect the inter-individual variability in finger skin friction. A 150 g custom-made iron cube (31 × 31 × 31 mm) served as an object, and it was placed on the 7.5 cm-height support, 30 cm away and in the midsagittal plane to the participants. Verbal instructions were provided requesting participants to grip the cube with minimal force by the pulp pinch using their dominant hands, lift it approximately 10 cm, and hold it for 10 s in space (Fig 1). The task was consecutively performed in the dominant hand for 10 trials with 5 s rest between each lift. Each participant repeated the same task twice, pre- and post-operatively. The averages of the total GF and COP trajectory over 10 trials were calculated for each patient.

### Materials

Two flexible and 0.1 mm-thick pressure sensor sheets (Pressure Mapping Sensor 5027, Tekscan, South Boston, MA, USA) were oppositely attached to the grip surfaces of the cube. The sensing area has a similar matrix height and matrix width of 27.9 mm, and each sheet had 1936 sensing elements (sensels) distributed over the same row and column quantity of 44. The density was 248 sensels per square cm, and the sensitivity ranged from 0 to 345 Kpa per sensel. Prior to each recording, equilibration and calibration were performed for each sensor sheet using the test instrument. I-scan 100 System (Nitta, Japan) was utilized to record pressure distribution data at 100 Hz frequency in 8-bit resolution. The data was saved as a movie file with a maximum of 36000 frames and 0.01 s in each frame period.

Two kinetic parameters of grip force control included total GF and the COP trajectory in the first-second and first five seconds. It is evident that the static phase is attained around 1 s after the object is held in the air [14]. As such, the first second duration is important to initially exhibit any meaningful differences. We inherited the finding to explore the total GF and COP trajectory at the first one second. Similar to our previous study, the GF trace was not stable with the bell-shaped curve (i.e., without peak GF in the lift phase) in many patients in the current study [15]. We therefore calculated the sum of the GF exerted and COP trajectory during the first five seconds. Consequently, the total GF and COP trajectory for the 1-sec and 5-sec were calculated from the sum of the pressure in all 1936 sensels during the first second and the fifth second, respectively. The COP is the center of all forces in the X- and Y-axes Cartesian coordinate system indicating the balance of the fingertip force on the sensor sheet. The COP displacement on the sensor sheets was recorded in each frame corresponding to 0.01 s as set prior. The calculation methodology for COP coordinates and distance of the COP displacement in a two-dimensional surface were similar to our previous study [11]. The parameters were collected pre- and postoperatively, and the data from the grip-and-lift task were investigated only in dominant hand.

### Clinical tests

Specific neurological tests for DCM, upper extremity functions, and grip-and-lift task were performed on the same day to follow the participants’ condition pre- and post-operatively. The severity of DCM was identified using the latest Japanese version of the JOA scale [16]. The level of severity was defined as severe if the JOA score is ranged from −2–8, moderate if the score is ranged from 9 to 13, and as mild if the score is ranged from 14 to17 [17]. In addition, following our previous study, we selected JOA upper extremity function (JOA-UEF) as the sub-score for upper extremity motor function [10]. The minimum JOA-UEF score is 0, and the maximum score is 4.

The following hand functions were assessed in the dominant hand pre- and post-operatively. The maximum pinch strength between the thumb and index finger with pulp pinch type was measured with a JAMAR Hydraulic Pinch Gauge (Petterson Medical, Warrenville, IL, USA). The maximum power of the hand grip was averaged from the three consecutive grips using a digital Smedley Grip Tester (Gopher, Owatonna, MN, USA). In the 10-s G&R test, participants were asked to perform full grip-and-release with the fingers as quickly as possible with the forearm in pronation. The number of cycles was counted within 10 seconds. Hand dexterity function was assessed by the three last subtests (numbers 8, 9, and 10) of the Simple Test for Evaluating Hand Function (STEF) in accordance with our previous studies [10,15]. The total time to complete three subtests was used as the total STEF. The cutaneous pressure threshold of the thumb and index finger pads was investigated using Semmes-Weinstein Monofilaments, which apply a standard target force of 0.07, 0.4, 2.0, 40, or 300 g. The lowest force recognized was considered normal, and the higher forces indicated, the more severe in sensory disturbance. In addition, static two-point discrimination (2PD) of the thumb and index finger was assessed using Touch-Test**®** Two Point Discriminator. One and two points with various standard testing intervals were consecutively applied to each finger pad in random order. Moreover, the Disabilities of the Arm, Shoulder, and Hand Questionnaire (DASH), a 30-item patient-reported outcome measure was employed to evaluate the symptoms and functionality of the complete upper limb. The total DASH disability/symptom score was used for statistical analysis.

### Statistical analysis

The Shapiro-Wilk test was performed to evaluate the normal distribution of data. As a result, normal distribution was confirmed in all kinetic parameters of the grip-and-lift task in both 1- and 5-sec, JOA total, JOA-UEF, pinch/grip strength, 10-s G&R test, and the cutaneous pressure threshold. However, normality was not satisfied in the STEF, 2PD, and DASH. Differences between pre- and post-operative variables with normal distribution were examined using paired t-test. Wilcoxon signed-rank test was performed to compare non-distributed normality data pre- and postoperatively. Associations between individual finger GF and COP in the pre-operative 1-sec with post-operative upper extremity functional tests were analyzed using Pearson’s correlation. To determine which preoperative variable best predicted the outcome of the decompressive surgery, a stepwise multiple regression analysis was used with post-operative JOA as the dependent variable, and preoperative 1-sec COP and GF of the index finger, grip strength, pinch strength, 10-s G&R test, STEF, 2PD, and DASH as the explanatory variables. Adjusted R^2^ was used to determine the Goodness of fit of the model. The selected model is the one with the highest adjusted R^2^. The statistical software SPSS ver. 27.0.1 for Windows (IBM SPSS Statistics, IBM Corp.) was used for the analyses, and *p* values < 0.05 were considered significant.

## Results

Table 1 summarizes the characteristics of the participants. In addition, the average duration of follow-up was 29.4 ± 21.4 days. Most half of the patients with DCM were at mild level, and the other half were at moderate to severe. There were two participants (6.9%) whose level of severity was not identified due to the missing data.

**Table 1:** Characteristics of the participants.

Demographic	
Age (year)	70.2 ± 6.8
Sex (male/female)	20/9
Duration of symptoms (month)	21.7 ± 21.1
Level of severity	
Mild	12 (41.4%)
Moderate	14 (48.3%)
Severe	1 (3.4%)
N/A	2 (6.9%)

**Fig 2:**
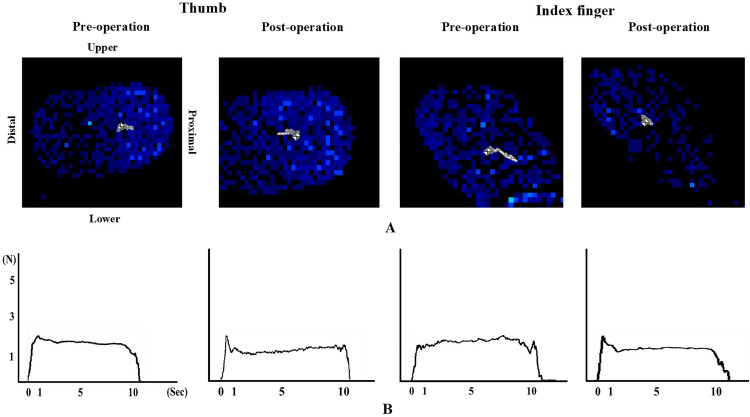
The center of pressure (COP) trajectories and grip force (GF) traces. The gray line appearing within the blue color area representing the contact of the finger pad indicates the COP trajectory (A) and GF profile (B) during a grip-and-lift trial in a same representative patient performing before and after surgery.

Table 2 demonstrates the results of comparison in clinical tests and grip-and-lift task between pre- and postoperative. In general, participants achieved significant improvement postoperatively. Both JOA total and JOA-UEF scores were significantly higher after surgery. Participants performed more time in the 10-s G&R test postoperatively, and the time taken to complete the STEF was postoperatively lower than that preoperatively. Cutaneous pressure threshold in both thumb and index finger significantly decreased postoperatively. The DASH motor score was also significantly lower postoperatively. However, there were no differences in grip/pinch strength and 2PD between pre- and postoperative. Most importantly, significant decrease was found in 1-sec COP trajectory in the index finger. The remaining kinetic parameters in the grip-and-lift task did not statistically change after decompression. Fig 2 shows the COP trajectories (A) and GF traces (B) by the thumb and index finger for a representative participant preoperatively and postoperatively.

**Table 2:** Summarized data from conventional clinical tests and grip-and-lift task.

		Pre-operation	Post-operation	*p* value
Conventional clinical tests				
JOA total^a^		12.4 ± 2.3	15.4 ± 1.7	**< 0.01**
JOA-UEF^a^		3.4 ± 0.9	4.0 ± 0.2	**< 0.01**
Pinch strength (Kg)^a^		4.0 ± 1.5	4.3 ± 1.4	0.18
Grip strength (Kg)^a^		25.6 ± 9.6	25.5 ± 9.0	0.85
10-s G&R test (time)^a^		15.6 ± 4.8	17.3 ± 4.2	**0.04**
STEF (sec)^b^		59.1 ± 35.7	48.8 ± 20.1	**0.01**
Cutaneous pressure threshold (g)^a^				
Thumb		1.0 ± 1.0	0.5 ± 0.7	**0.02**
Index finger		0.7 ± 1.0	0.1 ± 0.1	**< 0.01**
2PD (mm)^b^				
Thumb		6.1 ± 1.7	6.1 ± 2.1	0.39
Index finger		5.6 ± 1.9	5.4 ± 1.5	0.86
DASH motor^b^		25.4 ± 16.4	19.17 ± 10.7	**0.03**
Grip-and-lift task^a^				
COP (mm)				
1-sec	Thumb	14.4 ± 4.2	13.5 ± 4.5	0.45
	Index finger	15.3 ± 6.8	12.7 ± 4.0	**0.03**
5-sec	Thumb	37.2 ± 15.4	35.2 ± 13.0	0.57
	Index finger	38.1 ± 21.8	32.3 ± 13.4	0.14
GF (N)				
1-sec	Thumb	305.3 ± 219.6	348.9 ± 261.2	0.26
	Index finger	337.5 ± 285.9	363.6 ± 256.6	0.53
5-sec	Thumb	1775.3 ± 1823.2	1878.6 ± 1613.6	0.95
	Index finger	2037.5 ± 2205.7	2001.8 ± 1685.7	0.88

JOA: Japanese Orthopeadic Association; JOA-UEF: Japanese Orthopedic Association Upper Extremity Function; G&R: Grip and Release test; STEF: Simple Test for Evaluating Hand Function; 2PD: Two-point Discrimination; DASH: Disabilities of the Arm, Shoulder and Hand; COP: Center of Pressure; GF: Grip Force.

^a^ Paired t-test; ^b^ Wilcoxon signed-rank test.

Values in bold indicate significant difference.

**Fig 3:**
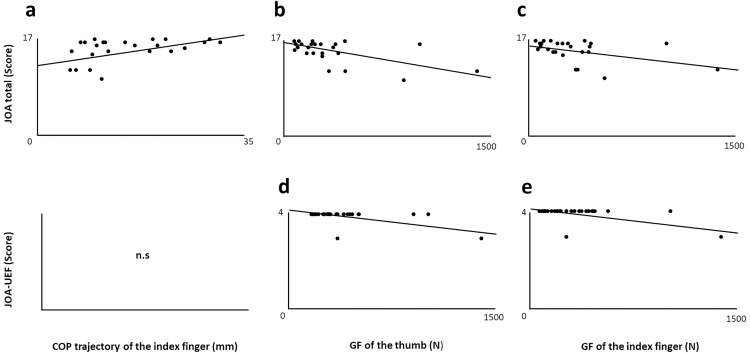
Correlations between the pre-operative 1-sec COP/GF and post-operative JOA total/UEF.

The results of correlations between the pre-operative 1-sec COP and GF with post-operative clinical tests in patients with DCM are presented in Table 3 and Fig 3. Pre-operative 1-sec COP of the index finger was positively correlated with postoperative JOA total score. Moreover, preoperative 1-sec GFs of the thumb and index finger were negatively correlated with both JOA total and JOA-UEF scores. As shown in Fig 3, the correlations with JOA total were stably distributed (a, b, and c). On the other hand, the correlations with JOA-UEF were unevenly distributed (d and e).

**Table 3:** Correlations between the pre-operative 1-sec COP and GF and post-operative upper extremity functional tests using Pearson’s correlation.

Post-operative Pre-operative		JOA total	JOA-UEF	Grip strength	Pinch strength	10-s G&R test	2PD Index finger	STEF	DASH motor
1-sec COP	Thumb	0.30	0.10	0.48*	0.19	0.33	0.03	−0.06	−0.28
	Index finger	0.44*	0.26	0.21	0.19	0.11	0.01	−0.01	−0.16
1-sec GF	Thumb	−0.55**	−0.48*	−0.39	−0.36	−0.38	0.34	0.24	0.49*
	Index finger	−0.43*	−0.48**	−0.35	−0.24	−0.42*	0.35	0.25	0.42*

COP: Center of Pressure; GF: Grip force; JOA: Japanese Orthopedic Association; UEF: Upper extremity function; G&R test: Grip and release; 2PD: Two-point discrimination; STEF: Simple Test for Evaluating Hand Function; DASH: Disabilities of the Arm, Shoulder and Hand.

*Correlation is significant at less than 0.05; **Correlation is significant at less than 0.01.

Values in bold indicate significant correlation.

To further investigate whether or not any kinetic parameters in 1-sec could predict the outcome of the surgery using JOA total score in addition to clinical upper extremity functional tests, a stepwise multiple regression was performed with three models (Table 4). The first model with only conventional clinical tests showed that pre-operative 2PD of the index finger could predict the postoperative JOA total score (*β* = −0.58, *p* < 0.01); the adjusted *R*^2^ value was 0.23. Model 2, in which preoperative 1-sec COP of the index finger was added in addition to the independent variables used in the first model, revealed that postoperative JOA total score could be better predicted by preoperative 2PD (*β = *−0.56, *p* < 0.01) and 1-sec COP trajectory of the index finger (*β =* 0.40*, p* = 0.03) with the higher adjusted *R*^2^ at 0.44. In Model 3, the 1-sec GF was included instead of preoperative 1-sec COP of the index finger as in Model 2, the result showed that pre-operative 2PD (*β =* −0.54*, p* < 0.01) and preoperative 1-sec GF (*β =* −0.38*, p* = 0.03) were less predictable for postoperative JOA total score at 0.42 of adjusted R^2^ value.

**Table 4:** Results of multiple regression analysis for predicting post-operative JOA total score.

	Dependent variable	Adjusted *R*^*2*^	Explanatory variable	Standardized β	*p* value
Model 1: conventional clinical tests	Post-operative JOA total	0.23	Pre-operative index 2PD	−0.58	< 0.01
Model 2: conventional clinical tests + 1-sec COP	Post-operative JOA total	0.44	Pre-operative index 2PD Pre-operative index 1-sec COP	−0.56 0.40	< 0.01 0.03
Model 3: conventional clinical tests + 1-sec GF	Post-operative JOA total	0.42	Pre-operative index 2PD Pre-operative index 1-sec GF	−0.54 −0.38	< 0.01 0.03

COP: Center of Pressure; GF: Grip force; JOA: Japanese Orthopedic Association; 2PD: Two-point discrimination.

## Discussion

We found that patients with DCM receiving decompressive surgery showed not only improvement in conventional clinical tests but also COP trajectory of the index finger in the first second of holding. Moreover, first second preoperative COP trajectory was correlated with the postoperative JOA total score. Adding COP trajectory to a set of common clinical tests could be more predictive of the surgical outcomes in DCM.

It is worth to emphasize that the COP trajectory of the index finger within the first second was significantly decreased after surgery (Table 2), implying the improvement of GF control after decompression of the cervical spine. Exploring deeper into the function of the fingers in grasping, studies evaluating spatial stability have initially demonstrated its usefulness in manipulating handheld objects [9,11]. With high-resolution sensors available, a sensor sheet is able to qualitatively capture even with minimal forces applied, as well as the smallest changes in deviations of force directions that are impossible to observe with bare eyes. In contrast to the conventional clinical tests, those kinetic measurements compromise to provide an insight into the fundamental dysfunction of individual finger at the most dexterous level. Based on these observations from previous studies and our results, we assume that the improvement will be equivalently observed in the kinetic assessment.

The improvement of kinetic analysis was only found in the COP trajectory, not in the total GF. In other words, although the post-operative COP trajectory improved in patients with DCM, the total GF remained unchanged. Our first hypothesis was partly supported. One underlying mechanism of this discrepancy could be because of the same experimental procedure when subjects were asked to perform with minimal force as far as possible. Even if the minimal force exerted were the same, its balance could still be influenced due to the inability to maintain proper finger posture on the contact surfaces and muscle incoordination, causing an altered the length of COP trajectory. Identified observations were found in stroke patients when object slippage could happen with the inappropriate force direction at the same force magnitude [18]. Additionally, children with severe developmental coordination disorder showed an irrelevance between COP trajectory and GF variability [19]. Our finding further contributed to the assertion that investigating the ability to hold an object stable requires a combination of GF magnitude and direction to accurately understand kinetics changes [11]. Thus, we concluded that the equating of the two kinetic values should also not be considered when evaluating DCM patients.

Contrary to our expectations, the postoperative COP trajectory decreased only in the index finger at the time of follow-up (Table 2), revealing a distinct recovery progression between the thumb and index finger. Ideally, proper coordination and balance of muscle activation allow the thumb and index finger to apply equal normal force magnitude and direction [20] and a force couple is considered [21]. Moreover, it is indicated that improvement in spasticity and muscle power after cervical spine decompression benefits early recovery in hand function [7]. Prior to the experiment, we therefore expected that the COP trajectory in both the thumb and index finger would improve after surgery. However, only the index finger COP showed postoperative improvement. This early recovery of the index finger compared to the thumb can be explained by the functional role of these fingers in precision grip. In a precision grip, the index finger engages in a single flexion to decrease the grip aperture, while the thumb performs a complex movement for opposition consisting of flexion and abduction at its joints [22]. Haggard and Wing identified the roles of the thumb and index finger during grasping. As such, the thumb takes the role of guiding the hand to the object while the index finger is responsible for closing the grip aperture and securing grasp safety [23]. This pattern was also found in monkeys with C4-5 lesions that succeeded in picking a piece of food by flexion at the index finger’s proximal interphalangeal joint and thumb opposition movement [24]. Moreover, the index finger then synchronizes with the object on one side as a stable base for the thumb to oppose and exert force on the other gripping side [25]. Based on these previous studies, the index finger with a single flexing function is reasonable to show early postoperative recovery compared to the thumb with a complex function. Our finding suggests a stepwise recovery sequence in precision grip, which contributes to monitoring postoperative changes based on the different functions of each finger and selecting appropriate rehabilitation.

Pre-operative GF was correlated with both post-operative JOA total and JOA-UEF (Fig 3 and Table 3). On the other hand, interestingly, GF was not correlated with grip or pinch strength. It implies that GF control reflects the comprehensive sensori-motor integration process, not indicates muscle strength itself. This view is consistent with our previous studies that reported the correlations between GF and sensori-motor deficits [9,10]. Taken together, it is reasonable to assume that the usefulness of pre-operative kinetic evaluation is not to predict motor function itself, but to know the overall clinical status after surgery. On the other hand, it is worth noting that the correlations with JOA-UEF were unevenly distributed (Fig 3. d and e), implying the possibility of ceiling effect in JOA-UEF after surgery. It is considered that JOA-UEF has a limitation of understanding the comprehensive sensori-motor integration process in DCM.

The model that relied solely on conventional clinical tests, utilizing 2PD as an explanatory variable, demonstrated the least effectiveness in predicting the post-operative JOA total score. Interestingly, an additional model for predicting the post-operative JOA total score incorporating COP trajectory had the largest adjusted R^2^ compared with conventional clinical tests (Table 4). Our second hypothesis was also partly supported. The 2PD was selected as one of the essential clinical tests in our current research because of its central mechanisms on sensory function. The 2PD is a sensory discriminative modality that conveys information regarding an individual’s spatial acuity. When the patients cannot discriminate one or two points, inhibitory mechanisms within the central nervous system, encompassing the spinal cord, subcortical structures, and cerebral cortex, play a significant role in the process of 2PD [26]. The DCM patients have direct compression on the cervical spinal cord regions. Therefore, the mechanism of the condition is thought to have a major impact on the patient’s ability to discriminate between two points. However, this lack of predictive capability may be attributed to the fact that 2PD solely measures superficial tactile acuity [27,28], while the JOA scale encompasses both motor and sensory functions. This might cause a lower prediction of neurological recovery. In contrast, a notable post-operative enhancement was recorded exclusively in the COP trajectory (Table 2), underscoring the relevance of COP in assessing surgical outcomes in DCM. Consequently, it is reasonable to consider the combined assessment of the COP trajectory, evaluating finger motor coordination during precision grasp, and 2PD, evaluating sensory dysfunction, as a vital predictor for neurological recovery in DCM.

Several limitations of the present study should be acknowledged. Firstly, we did not investigate symptoms at each spinal cord level, because all participants underwent decompression surgery at C3-7 level. However, DCM symptoms are related to compression levels and areas of the spinal cord [29]. In the future, we need to use MRI to examine the relationship between the spinal cord compression level and area and grip force control. Secondly, the manual dexterity problems in DCM stem from a combination of neurological impairments affecting strength, sensation, and complex hand function [30]. Especially, weakness in the intrinsic hand muscles disrupts fine motor tasks, while sensory deficits impair precise object manipulation. More comprehensive evaluation tests would have been desirable to prove that total GF and COP trajectory are valid methods for assessing upper limb function in DCM. Thirdly, we only analyzed the GF control of the dominant hand. Since DCM affects both sides of the body, focusing only on the dominant hand may lead to an incomplete understanding of a patient’s overall functional capabilities. To gain a more comprehensive understanding of GF control, a future study evaluating object manipulation with both hands simultaneously should be considered. Fourthly, in terms of study design, the sample size was small. The study was conducted immediately after the global COVID-19 pandemic, making it extremely challenging to recruit adequate participants and conduct measurements across multiple sites. As a result, the sample size remained limited. In the future work, organizational-level administrative support will be essential for increasing the sample size. Fifthly, the patient’s recovery at the consecutive follow-up periods was not monitored. Previous studies have explored hand function recovery in various time points such as 24 hours, 1 week, or 3 months [31–33]. Additional measurement points need to be considered to identify a detailed analysis of the time course of recovery of GF control. Lastly, although we found correlations of COP/GF with conventional upper extremity functional tests, the validity of COP/GF in DCM was not comprehensively discussed. To more clearly evaluate the usefulness of these kinetic parameters, studies based on the specific feature of DCM need to be considered in the future.

In conclusion, our study demonstrated that recovery of COP trajectory in patients with DCM can be achieved after decompressive surgery, and is related to neurological function. Moreover, the model including COP trajectory provided the highest adjusted R^2^ during precision grip task and was ideally optimal for predicting the surgical outcomes. This new knowledge may enrich our understanding of hand function recovery in patients with DCM and support selecting rehabilitative programs for hand functions.

## Supporting information

S1 Data Data from conventional clinical tests and grip-and-lift task. (XLSX)
